# Applying nomograms based on the surveillance, epidemiology and end results database to predict long-term overall survival and cancer-specific survival in patients with oropharyngeal squamous cell carcinomas: A case–control research

**DOI:** 10.1097/MD.0000000000020703

**Published:** 2020-07-24

**Authors:** Fengze Wang, Jiao Wen, Xinjie Yang, Tingting Jia, Fangchong Du, Jianhua Wei

**Affiliations:** a State Key Laboratory of Military Stomatology & National Clinical Research Center for Oral Diseases & Shaanxi Clinical Research Center for Oral Diseases, Department of Oral and Maxillofacial Surgery School of Stomatology, The Fourth Military Medical University, Xi’an, China; b Department of Stomatology, The eighth medical center of Chinese PLA General Hospital, Beijing, China; c State Key Laboratory of Military Stomatology & National Clinical Research Center for Oral Diseases & Shaanxi Engineering Research Center for Dental Materials and Advanced Manufacture, Department of Anesthesiology, School of Stomatology, The Fourth Military Medical University, Xi’an; d Department of Stomatology, The Chinese PLA General Hospital, Haidian District, Beijing, China.

**Keywords:** calibration curve, cancer-specific survival, C-index, nomogram, oropharyngeal squamous cell carcinoma, overall survival, surveillance, epidemiology and end results

## Abstract

Few models regarding to the individualized prognosis assessment of oropharyngeal squamous cell carcinoma (OPSCC) patients were documented. The purpose of this study was to establish nomogram model to predict the long-term overall survival (OS) and cancer-specific survival (CSS) of OPSCC patients. The detailed clinical data for the 10,980 OPSCC patients were collected from the surveillance, epidemiology and end results (SEER) database. Furthermore, we applied a popular and reasonable random split-sample method to divide the total 10,980 patients into 2 groups, including 9881 (90%) patients in the modeling cohort and 1099 (10%) patients in the external validation cohort. Among the modeling cohort, 3084 (31.2%) patients were deceased at the last follow-up date. Of those patients, 2188 (22.1%) patients died due to OPSCC. In addition, 896 (9.1%) patients died due to other causes. The median follow-up period was 45 months (1–119 months). We developed 2 nomograms to predict 5- and 8- year OS and CSS using Cox Proportional Hazards model. The nomograms’ accuracy was evaluated through the concordance index (C-index) and calibration curves by internal and external validation. The C-indexes of internal validation on the 5- and 8-year OS and CSS were 0.742 and 0.765, respectively. Moreover, the C-indexes of external validation were 0.740 and 0.759, accordingly. Based on a retrospective cohort from the SEER database, we succeeded in constructing 2 nomograms to predict long-term OS and CSS for OPSCC patients, which provides reference for surgeons to develop a treatment plan and individual prognostic evaluations.

## 1 Introduction

Oropharyngeal squamous cell carcinoma (OPSCC) is the most common malignant head and neck cancer^[1]^ and it is mainly located in the pharynx, tongue root, pharyngeal tonsil, and soft palate. The annual incidence of oropharyngeal cancers is approximately 400,000 new OPSCC patients in the world, and the incidence of OPSCC has increased sharply in developed countries and nearly 46,000 cases in the United States.^[2–4]^ Currently, National Comprehensive Cancer Network (NCCN) guidelines recommend applying the American Joint Committee on Cancer (AJCC) Staging Manual (7th edition) to evaluate the prognosis of OPSCC patients.^[5,6]^ However, the prognosis of OPSCC patients is influenced by numerous factors, such as age, cigarette and alcohol consumption, tumor site, TNM stage, radiation, and human papilloma-virus (HPV).^[1,7]^ Thus, the consideration of additional relevant elements should provide a more accurate and credible prediction of prognosis than the AJCC staging system. Therefore, we sought to establish a “nomogram” to identify additional relevant factors including age, sex, tumor site, race, different origin, grade, T stage, N stage, M stage, surgery, and radiotherapy to perform a comprehensive analysis. To verify precision and credibility, many researchers recommend using the split-sample method and bootstrap to evaluate a given model.^[8–11]^ Specifically speaking, the nomogram is internally validated by bootstrap re-sampling and externally validated by appraising model's accuracy in split-sample cohorts.^[12]^ The size of the validation cohort (the split-ratio) depends on the coherence and accuracy between the predicted and actual outcomes rather than a fixed value.^[13]^ The accuracy of the nomograms is determined via C-indexes and calibration curves.

A nomogram is an accurate scoring and graphical instrument that can convert the results of multivariate Cox regression into an understandable linear graph. Nomograms are widely used to assist doctors in formulating a therapeutic regimen and have been shown to predict the prognosis of several cancers, including adenoid cystic carcinoma,^[14]^ hepatocellular carcinoma,^[15,16]^ gastric cancer,^[17]^ head and neck cancers,^[18]^ nasopharyngeal cancer,^[19]^ and breast cancer.^[20]^ Most importantly, the application of a nomogram in the early detection of prostate cancers has been included in the NCCN guidelines.^[21]^ Additionally, it is worth noting that the American Joint Committee on Cancer Eighth Edition Cancer Staging Manual indicated that a future version will embrace nomograms and individualized treatment strategies.^[22]^ One previous study used a nomogram to assess the prognosis and progression of OPSCC.^[23]^ However, this research didn’t take race, origin, pathological grade, and surgery into account to predict the overall survival (OS) and cancer-specific survival (CSS). In our research, we collected the detailed OS and CSS information of the OPSCC patients. We used the Kaplan–Meier univariate and Cox Proportional Hazard Model multivariate survival analysis to determine the final independent risk clinicopathological parameters influencing the prognosis (*P* < .05). Hence, we sought to establish an OPSCC nomograms to predict long-term OS and CSS based on relevant clinicopathological parameters mentioned above by means of R software R.3.2.4 (Lucent, New Jersey, USA), which should help doctors develop rational and personalized treatments.

## 2 Methods

### 2.1 Patient clinical data collection

All 10,980 OPSCC patients from the years 2004 to 2012 were included in this retrospective research, and the clinical data were gathered from the SEER program of the National Cancer Institute.^[24]^ We collected and sorted the detailed clinicopathological parameters, including age, sex, tumor site, race, ethnic origin, pathological grade, surgery or no surgery, radiation or no radiation, T stage, N stage, and M stage (Table 1). The pathological grade consists of Grade I, II, III, IV. Grade I, II, III, IV represent well differentiated, moderately differentiated, poorly differentiated and undifferentiated respectively. Based on the split-sample method discussed in the Introduction, we randomly split the 10,980 OPSCC patients into a modeling cohort and an external validation cohort. The modeling cohort consisted of 9881 OPSCC patients to build a nomogram model. Another 1099 OPSCC patients were included in the external validation cohort to verify the performance and credibility of nomogram model. The study was approved by the Ethical Review Committee of the Fourth Military Medical University.

**Table 1:**
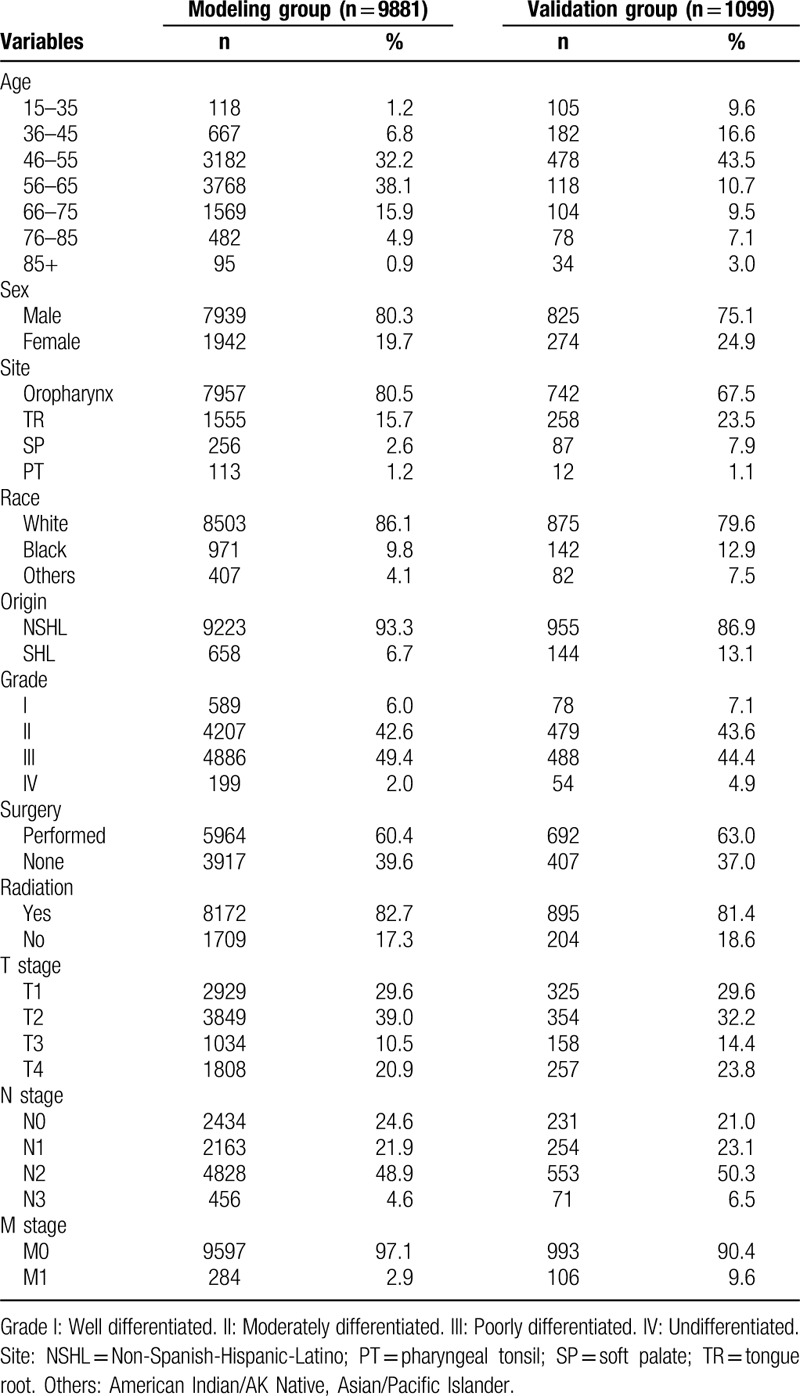
Patients’ clinicopathological data.

### 2.2 Survival analysis

We concentrated on the indicators of OS and CSS to assess the prognosis of modeling cohort. OS was defined from the date of diagnosis to death from any reason or censored if patients were alive at the last follow-up. CSS was calculated from diagnosis to death due to OPSCC or censored if patients were alive or dead because of other causes.

We conducted univariate survival analysis using a Kaplan–Meier and log-rank test. The variables that were statistically significant were included into the multivariate Cox Proportional Hazards analysis to confirm the independent prognostic factors from indicators, such as age, sex, tumor site, race, ethnic origin, pathological grade, surgery, radiation, T stage, N stage, and M stage (Tables 2 and 3 ). *P* < .05 was considered statistically significant.

**Table 2:**
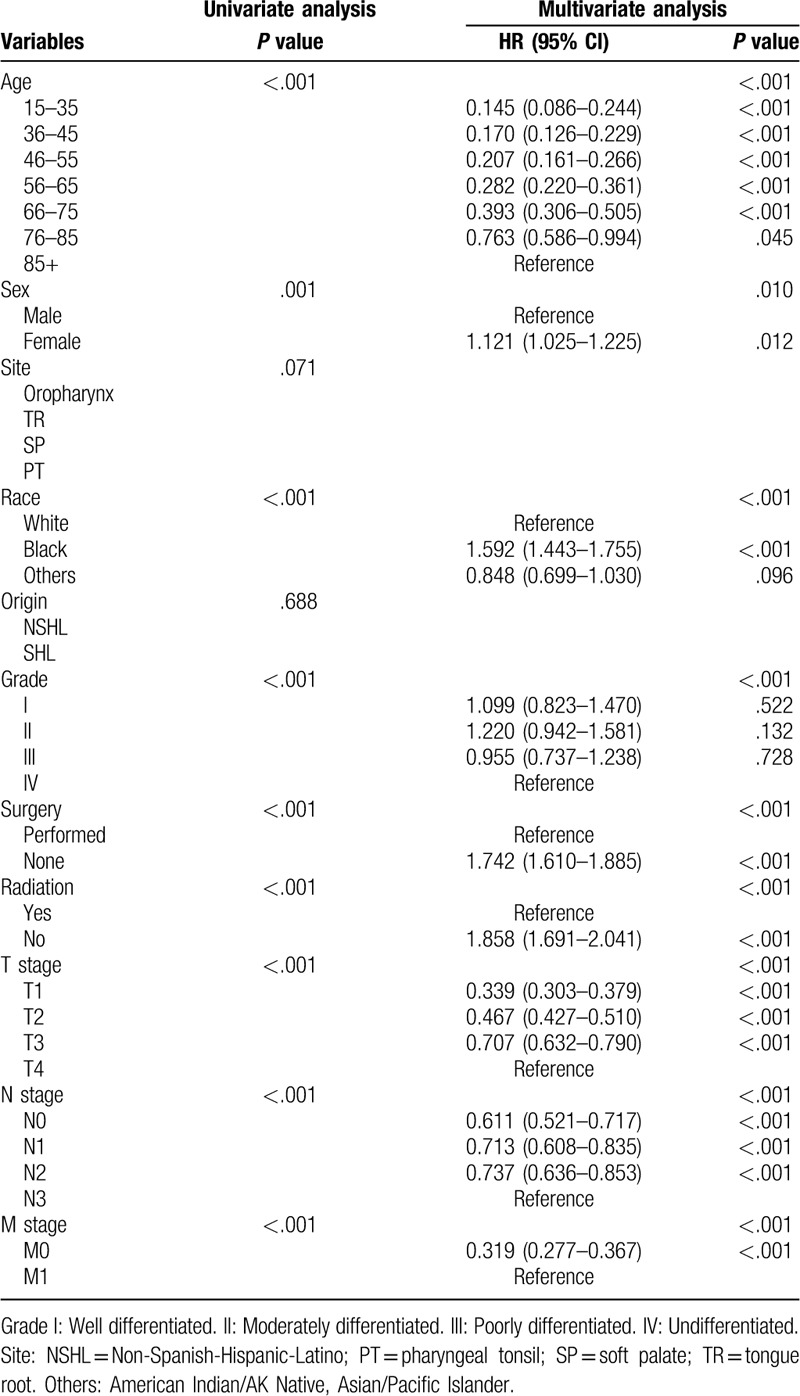
Univariate and multivariate analyses of OS in nomogram cohort.

**Table 3:**
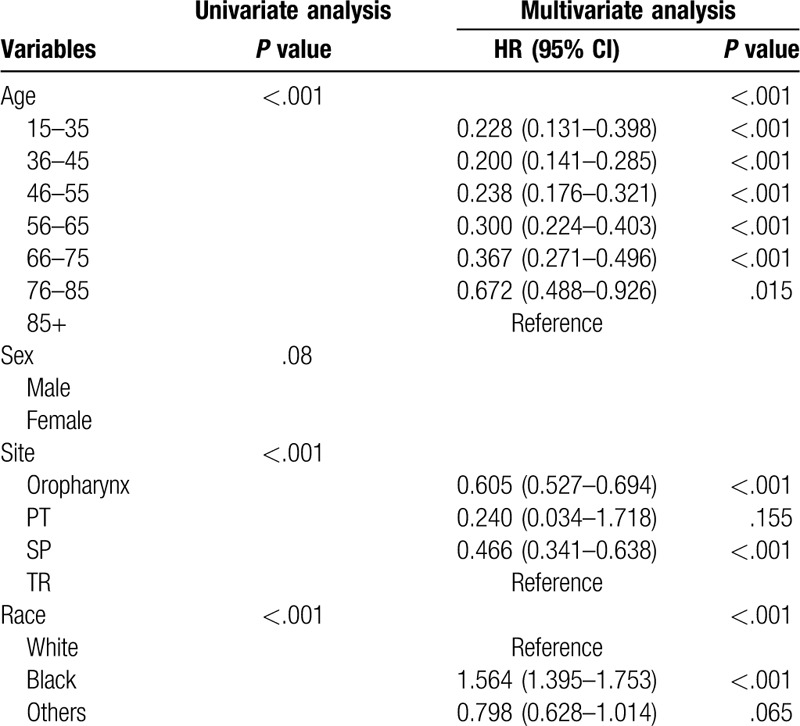
Univariate and multivariate analyses of CSS in nomogram cohort.

**Table 3 (Continued):**
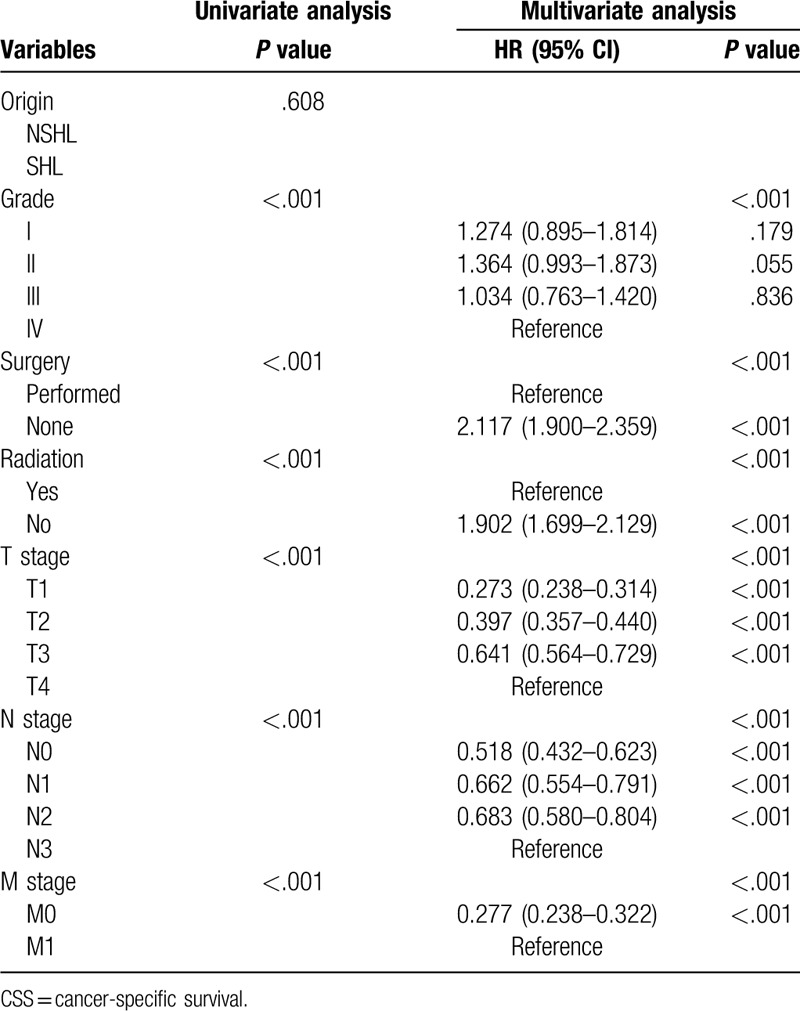
Univariate and multivariate analyses of CSS in nomogram cohort.

### 2.3 Nomogram development

After conscientious survival analysis using SPSS 21.0 (IBM, Armonk, USA) for Windows software or the rms package of R version 3.2.4 software, we used the independent prognostic indicators for establishing a nomogram.^[11,25]^ The nomogram transformed the clinicopathological data into visual graphics.

### 2.4 Nomogram validation

The nomogram was required to validate its accuracy through internal and external validation conducted by 1000 times bootstrapping and 10-fold cross-validation measures. The predict reliability was examined in concordance indexes (C-index) and calibration plot.^[11]^ The C-index was used to appraise the difference between predicted and actual situations.^[11]^ The C-index results were acquired through the “rcorrcens” command in R software. Additionally, the calibration plot was composed of two lines: one was a 45° diagonal line representing a reference line, and the other line was the actual line. The distance between the 2 lines reflected the precision of the nomogram. The calibration plots were obtained through the “calibrate” command in R software. All statistical analyses were performed adopting a 2-sided *P* value, and *P* < .05 was considered to be statistically significant.

## 3 Results

### 3.1 Patient clinicopathological data

After applying a strict filter, 9881 and 1099 OPSCC patients from SEER database were included in the modeling and validation cohorts respectively, using the popular and reasonable random split-sample method (the split ratio was 9:1). In the modeling cohort, the patients’ ages ranged from 15 to 98 years (median, 57). Of these 9881 OPSCC patients, 7936 (80.3%) were men. In total, 8503 (86.1%) patients were white, and 9223 (93.3%) patients were non-Spanish-Hispanic-Latino. Of the tumor locations, 7957 (80.5%) cases were primarily located on the oropharynx and 1555 (15.7%) were found on the tongue base. Additionally, 5085 (51.4%) were poorly defined or undifferentiated. Of the studied cases, 5964 (60.4%) received surgery and 8172 (82.7%) received radiotherapy. The proportion of T1–T2 tumors was 68.6% (6778/9881). The N1–3 and M1 tumors accounted for 75.4% and 2.9% of all cases, respectively. The general data for the validation cohort are shown in Table 1.

According to the SAS variable “sur_time_mon” from the SEER database, we found that the median follow-up times for the modeling and validation cohorts were 45 months (1–119 months) and 57 months (1–119 months). According to the SAS variables “STAT_REC,” “VSRTSADX,” and “ODTHCLASS” in SEER database, we obtained accurate information on the outcomes for 10,980 OPSCC patients. A total, of 3084 (31.2%) patients in the modeling cohort were deceased at the last follow-up date. Of those patients, 2188 (22.1%) patients died due to OPSCC. Additionally, 896 (9.1%) patients died from causes other than OPSCC.

### 3.2 Survival analysis and nomogram establishment

The result of the survival analysis with regard to OS and CSS is shown in Tables 2 and 3 . For the modeling cohort, the results of the univariate Kaplan–Meier survival analysis revealed that age, sex, race, pathological grade, surgery, radiation, T stage, N stage, and M stage were relevant factors influencing OS (*P* < .05). Multivariate Cox Proportional Hazards analysis showed that all the above elements from the univariate analysis were independent prognostic indicators (*P* < .05), which were shown in Table 2.

**Figure 1:**
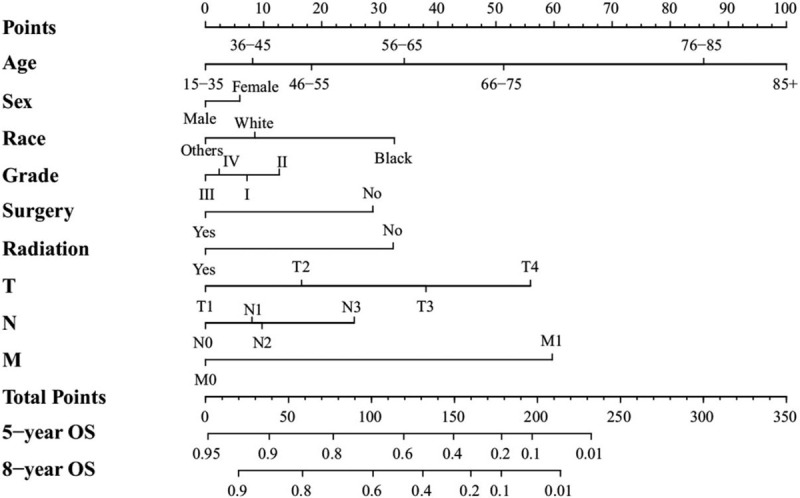
Nomogram predicting 5-year and 8-year OS. Others: American Indian/Alaska Native/Asian or Pacific Islander. Grade I: well differentiated. II: moderately differentiated. III: poorly differentiated. IV: Undifferentiated.

Thus, these factors were included to construct a nomogram to predict 5- and 8-year OS (Fig. 1). The prognosis survival analysis of OS was conducted using SPSS 21.0 (IBM, Armonk, USA) software for Windows.

**Figure 2:**
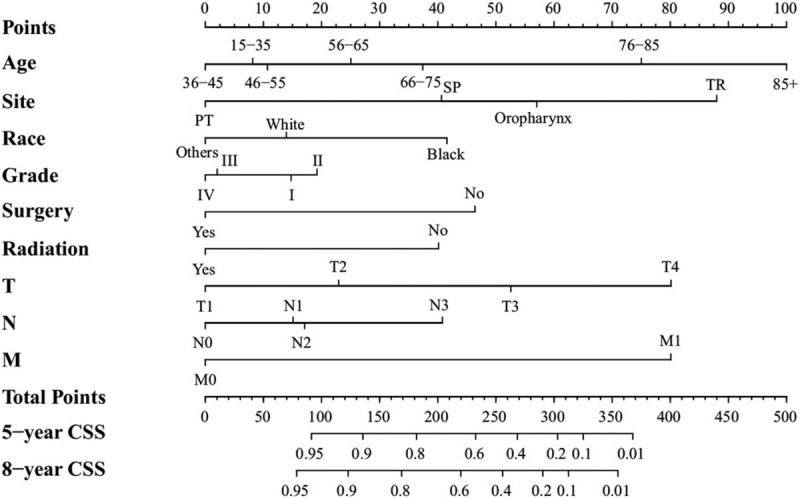
Nomogram predicting 5-year and 8-year CSS. Others: American Indian/Alaska Native/Asian or Pacific Islander. Grade I: well differentiated. II: moderately differentiated. III: poorly differentiated. IV: undifferentiated. PT: pharyngeal tonsil. SP: soft palate. TR: tongue root.

The results identified that age, race, tumor site, pathological grade, surgery, radiation, T stage, N stage, and M stage as independent factors influencing CSS (Table 3 ). Furthermore, these factors were used to establish another nomogram to forecast the 5-year and 8-year CSS (Fig. 2).

### 3.3 Nomogram validation

**Figure 3:**
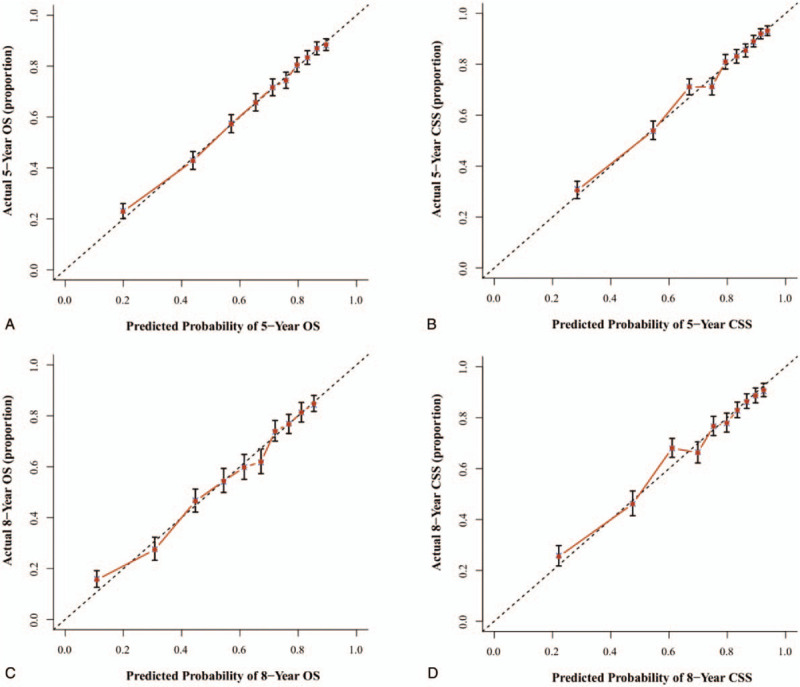
Internal validation via calibration curves for 5-year and 8-year OS (A, C) and 5-year and 8-year CSS (B, D). The 45° line embodys an perfect match between the actual survival (*y* axis) and nomogram-predicted survival (*x* axis). The perpendicular line means 95% confidence intervals. CSS = cancer-specific survival; OS = overall survival.

**Figure 4:**
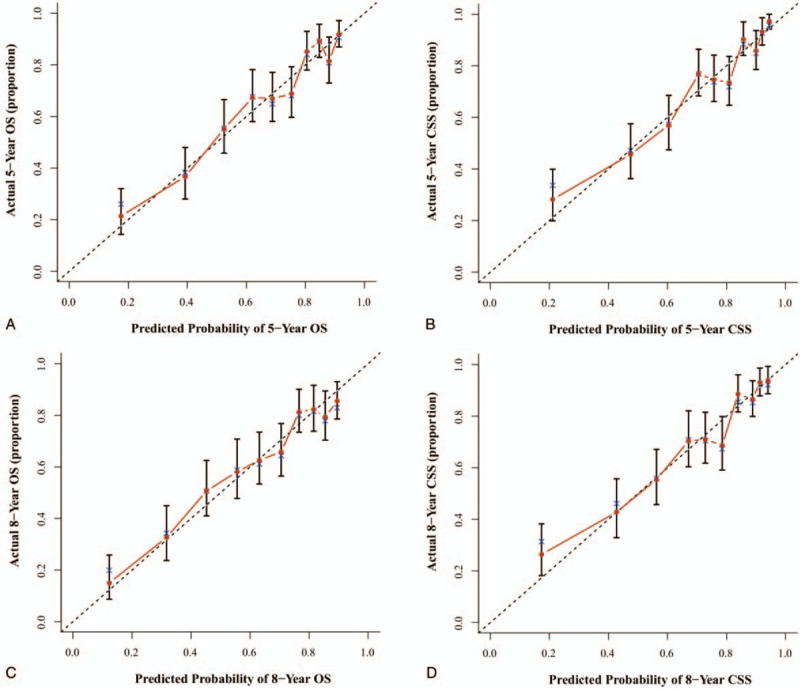
External validation via calibration curves for 5-year and 8-year OS (A, C) and 5-year and 8-year CSS (B, D). The 45° line embodys an perfect match between the actual survival (*y* axis) and nomogram-predicted survival (*x* axis). The perpendicular line means 95% confidence intervals. CSS = cancer-specific survival; OS = overall survival.

The nomogram's credibility are internally validated by 1000 times bootstrap re-sampling and externally validated by evaluating model's accuracy in split-sample cohort of 1099 patients. The concordance index (C-index) and calibration curves were used to assess the precision of the nomograms. Because the value of C-index was >0.7, the predicted OS and CSS were consistent with the actual OS and CSS. Our results of internal validation showed that the C-index values of OS and CSS were 0.742 and 0.765, respectively. External validation revealed that the C-index of OS and CSS were 0.740 and 0.759, respectively. Moreover, the internal and external calibrations were close to the 45° ideal straight line (Figs. 3 and 4).

## 4 Discussion

Due to the combined effects of clinicopathological factors, an increasing number of patients have been diagnosed with OPSCC.^[26]^ Generally, surgery, radiation and chemotherapy are familiar therapeutic methods for OPSCC, which would influence the patients’ prognosis to a great extent.^[27]^ Additionally, other relevant factors including age, sex, smoking, TNM stages also affected the prognosis of patients with OPSCC. In order to provide individualized, patient-specific prediction of prognosis, we have developed and validated two nomograms to forecast 5- and 8-year OS and CSS of patients with OPSCC via popular random split-sample method (split ratio = 9:1), which was been applied extensively.^[28]^ Nomogram could integrate the clinical and pathological factors together. Notably, the 8th Head Neck Cancer AJCC staging system showed that in the future version they would incorporate the nomogram to evaluate the prognosis.

We calculated the estimated OS and CSS by means of Kaplan–Meier method. After the Kaplan–Meier univariate and Cox multivariate survival analysis, we obtained the independent prognostic risk factors and established 2 nomograms. The nomograms were well validated internally and externally. The C-index of all nomograms were >0.7, and there was a good consistency between the calibration curve and the 45° straight line, showing a potential advantage than Shoultz-Henley model which was short of validation.^[29]^ There had quantitative axis for each index, corresponding to the axises representing 5- and 8-year OS and CSS (see Figs. 1 and 2). The more left the axis was, the higher the survival rate was. We found that OS and CSS gradually declined after the age of 55. Therefore, the age groups of “15 to 35” and “36 to 45” were at the far left of the age axises, demonstrating the best OS and CSS respectively. Many studies have found that age was a significant element influencing survival.^[26,27,30,31]^ Another study showed that patients <45 years old had the best CSS compared with the age groups of “45 to 64” and “>65”, conforming to our research.^[26]^ Compared with white patients and patients of other races, black patients have demonstrated relatively lower survival, which is in agreement with the outcomes of the current research.^[32]^ As a mechanism, one study hypothesized that melanin might contribute to tumorigenesis and cancer development.^[18]^ Notably, the patients with pathological grades III and IV disease showed improved OS and lower cancer-specific death compared with patients with grades I and II disease (Table 3 ). Moreover, the results were also verified in the nomograms constructed. This may be because that radiotherapy and surgery were used to treat the higher-grade patients primarily. T stage, N stage were also the significant predictive factors influencing the prognosis.^[33,34]^

The procedure of nomograms to predict the 5- and 8- year OS and CSS was simple and feasible. We selected clinicopathological factor sub-categories according to personalized conditions and constructed a vertical line to the point axis. Then, we added all the points acquired by each sub-category corresponding to the total points axis. Finally, we plotted vertical lines from the total points to the 5- and 8- year OS and CSS axis to obtain the predicted value. This process was completed using the “rms” package of R software.^[35]^ Additionally, we conducted both internal and external validation; the concordance indexes were all >0.7, and they matched well with the 45° straight line (Figs. 3 and 4). The nomogram was more accurate than TNM staging for predicting the prognosis of OPSCC. As an example, compare the following 2 types of T4N0M0 patients: type 1, a 50-year-old white patient with pathological grade III disease who received only surgery, and type 2, a 60-year-old black patient with pathological grade II disease underwent surgery and radiation. If we evaluate the prognosis of those 2 types of patients according to AJCC TNM classification,^[6]^ the 2 patients all belonged to stage IV, having the same outcome. Yet, the results were different using the nomogram. The 5-year predicted OS for type 1 and type 2 patient was 76% and 30% accordingly. Therefore, we included the independent prognostic factors into the model to construct more credible nomograms to predict the OS and CSS.

Our research had obvious strengths. First, we collected detailed and reliable information regarding OPSCC patients from the SEER database to guarantee the credibility of the results. The data came from 18 SEER registries located in 18 different states, which was a large-sample multi-center research. Secondly, our nomograms have potential advantage over previously published models for OPSCC. Shoultz-Henley et al^[29]^ had established a nomogram, but the model was neither internally nor externally validated. By contrast, we validated the our nomogram models via C-index and calibrations, showing a higher accurancy. Rios et al^[33]^ also constructed a nomogram, but our larger sample capacity and longer follow-up period allowed us to develop a separate nomograms about 5- and 8-year OS and CSS in patients with OPSCC. Karadaghy et al^[36]^ had developed prediction model using machine learning for 5-year overall survival. However, the main obstacles to the widespread application of this algorithm include convenience, regulatory, and financial considerations.

Our research had certain limitations. First, the SEER database didn’t include other significant prognostic factors, such as anemia,^[33]^ chemoradiation,^[37]^ and cigarette and alcohol consumption status,^[32]^ thrombocytosis.^[29]^ Thus, our nomograms lacked evaluation of the above elements. Also, we couldn’t assess the disease-free survival, progression-free survival and loco-regional control. Second, we couldn’t obtain the dose, cycle and type of radiotherapy to compare the survival differences between various radiation plans. Third, we couldn’t acquire the life habits of all the patients, which may influence the prognosis. Meanwhile, we couldn’t gain the TNM staging information before the year 2004.

In conclusion, we have constructed 2 successful nomograms forecasting 5- and 8- year OS and CSS via Cox regression. We also obtained favorable C-indexes through internal and external validation. These nomograms may provide surgeons with a reference to develop treatment plans and conduct individual prognostic evaluations, as the future will most certainly bring an era of personalized therapy.

## Acknowledgment

The authors express their gratitude to Hui Zhang for her guidance to data analysis.

## Author contributions

**Conceptualization:** Fengze Wang, Jiao Wen, and Jianhua Wei.

**Data curation:** Jiao Wen, Tingting Jia, Fangchong Du.

**Formal analysis:** Jiao Wen, Xinjie Yang, and Tingting Jia.

**Funding acquisition:** Jianhua Wei.

**Investigation:** Fengze Wang, Jiao Wen, Tingting Jia

**Methodology:** Xinjie Yang, Tingting Jia, and Fangchong Du.

**Project administration:** Fengze Wang, Jiao Wen, and Jianhua Wei.

**Resources:** Tingting Jia and Fangchong Du.

**Software:** Jiao Wen and Tingting Jia.

**Supervision:** Jianhua Wei.

**Writing – original draft:** Fengze Wang, Jiao Wen, Xinjie Yang, Tingting Jia, and Fangchong Du.

**Writing – review & editing:** Fengze Wang, Xinjie Yang, and Jianhua Wei.
